# Endothelial type I interferon response and brain diseases: identifying STING as a therapeutic target

**DOI:** 10.3389/fcell.2023.1249235

**Published:** 2023-09-14

**Authors:** Nádia Duarte, Abdul Muktadir Shafi, Carlos Penha-Gonçalves, Teresa Faria Pais

**Affiliations:** Instituto Gulbenkian de Ciência, Oeiras, Portugal

**Keywords:** endothelial cells, type I IFN, STING, brain, inflammation

## Abstract

The endothelium layer lining the inner surface of blood vessels serves relevant physiological functions in all body systems, including the exchanges between blood and extravascular space. However, endothelial cells also participate in innate and adaptive immune response that contribute to the pathophysiology of inflammatory disorders. Type I Interferon (IFN) signaling is an inflammatory response triggered by a variety of pathogens, but it can also be induced by misplaced DNA in the cytosol caused by cell stress or gene mutations. Type I IFN produced by blood leukocytes or by the endothelium itself is well-known to activate the interferon receptor (IFNAR) in endothelial cells. Here, we discuss the induction of type I IFN secretion and signaling in the endothelium, specifically in the brain microvasculature where endothelial cells participate in the tight blood-brain barrier (BBB). This barrier is targeted during neuroinflammatory disorders such as infection, multiple sclerosis, Alzheimer’s disease and traumatic brain injury. We focus on type I IFN induction through the cGAS-STING activation pathway in endothelial cells in context of autoinflammatory type I interferonopathies, inflammation and infection. By comparing the pathophysiology of two separate infectious diseases—cerebral malaria induced by *Plasmodium* infection and COVID-19 caused by SARS-CoV-2 infection—we emphasize the relevance of type I IFN and STING-induced vasculopathy in organ dysfunction. Investigating the role of endothelial cells as active type I IFN producers and responders in disease pathogenesis could lead to new therapeutic targets. Namely, endothelial dysfunction and brain inflammation may be avoided with strategies that target excessive STING activation in endothelial cells.

## 1 Introduction

The brain vasculature structure contributes to a particular environment essential for neuronal functioning. The selective permeability of the blood-brain barrier (BBB) protects the brain from neurotoxins while allowing the supply of nutrients via solute carriers and the elimination of by-products of brain metabolism via efflux transporters [reviewed in (Sweeney et al., 2019)]. BBB dysfunction underlies a large number of neurologic diseases, including those triggered by pro-inflammatory responses to viruses and to the malaria parasite (Patabendige and Janigro, 2023).

Type I interferons (IFNs) are inflammation mediators induced via activation of pathogen recognition receptors by microbial nucleic acids or by endogenous DNA misplaced in the cytosol (Wu and Chen, 2014). The type I IFN response is pleiotropic: antireplicative in viral infections, immunosuppressive in multiple sclerosis (Trinchieri, 2010) or autoinflammatory in interferonopathies (Crow et al., 2022). The multiple beneficial and deleterious roles of type I IFN in the immune response against pathogens is exemplified by SARS-CoV-2 infection (Sposito et al., 2021), and has long been known in the field of *Plasmodium* infection, which causes malaria (Sebina and Haque, 2018). Experimental and human data suggest that during infection progression, the time windows of type I IFN secretion, the innate immune receptors involved, and the cells-types activated, are main determinants of whether type I IFN responses will potentiate anti-viral or anti-parasitic mechanisms or instead heighten immunopathology and tissue damage events (Sebina and Haque, 2018; Chiale et al., 2022).

Here, we discuss the role of endothelial type I IFN response during disease. In the last 5 years, the induction of type I IFN via the cGAS-STING pathway was linked to alterations in the vasculature (vasculopathy) and inflammation of the blood vessels (vasculitis). Excessive type I IFN induction can be caused either by gain of function mutations in the STING gene such as in STING-associated vasculopathy with onset in infancy (SAVI), a type I interferonopathy or by STING activation during infectious diseases. STING activation in the brain endothelium leads to leukocyte recruitment and contributes to *Plasmodium-*induced cerebral vasculopathy (Pais et al., 2022). Furthermore, STING activation by mitochondrial DNA appears to be part of the lung vasculopathy caused by SARS-Cov-2 (Domizio et al., 2022). Thus, STING inhibition in endothelial cells may open new avenues for therapeutic intervention aiming at protecting the endothelium in several pathologies.

## 2 A brief overview of the brain vasculature

Endothelial cells (ECs) are in close contact with the bloodstream and constitute a selective barrier to circulating molecules, cells, and pathogens. In the luminal side, ECs expose a complex network of macromolecules, mostly negatively charged membrane-bound proteoglycans, glycolipids, and glycosaminoglycans that form the glycocalyx. The glycocalix contributes to cell signaling and ECs properties such as mechanotransduction and adhesion of cells and molecules to the endothelium (Reitsma et al., 2007; Krüger-Genge et al., 2019). On their basolateral surface, a glycoprotein basement membrane, produced by ECs themselves, delimits the surrounding tissues.

The term endothelium, originally coined by the Swiss anatomist Wilhem His in 1865, designates the inner layer of ECs that participate in many vascular physiological functions such as permeability, blood cell trafficking, nutrient and oxygen transport, hemostatic balance and control of vasomotor tone, as well as innate and adaptive immunity (Aird, 2007; Claesson-Welsh et al., 2021; Amersfoort et al., 2022). Currently, three main types of endothelium are considered (Aird, 2007; Hennigs et al., 2021; Gifre-Renom et al., 2022), the continuous endothelium, fenestrated endothelium and discontinuous endothelium. The most common in the brain is the continuous endothelium of capillaries (corresponding to 85% of the brain vessels length) (Sweeney et al., 2019). The microvascular endothelial cells are connected by tight junctions formed by transmembrane proteins such as claudin 5 (CLDN-5), occludins, junctional adhesion molecules and cytoplasmic adaptors, such as zona occludens proteins (e.g., ZO-1) (Sweeney et al., 2019). These tight junctions, together with the lack of fenestrations and low pinocytic activity, prevent the paracellular molecular passage, establishing a tight endothelial barrier against the flow of solutes and water-soluble molecules from the blood into the brain and contributing to the blood brain barrier (BBB). The BBB structure is further strengthened by the basement membrane and interactions between the endothelial cells and CNS cells, pericytes, astrocytes and microglia on the abluminal side. Functionally, this structure provides a neurovascular unit, which integrates vascular and neuronal activities in order to maintain brain homeostasis [reviewed in (McConnell and Mishra, 2022)]. These unique endothelial cell features are essential to protect the brain against harmful blood-borne factors and pathogens.

Site-specific endothelium structural properties are dynamically regulated during embryogenesis and postnatal period and can be modulated under pathologic conditions (Aird, 2007; Augustin and Koh, 2017; Potente and Mäkinen, 2017; Gifre-Renom et al., 2022). The development of the brain vasculature and the establishment of the BBB depend on the canonical Wnt/β-catenin pathway. Namely, the WNT7A/WNT7B ligands together with two membrane proteins, Reck (reversion inducing cysteine rich protein with kazal motifs) and Gpr124 (an orphan G protein-coupled receptor), promote the BBB formation and maintenance (Cho et al., 2017). Endothelial-specific Gpr124 deficient mice are more susceptible to stroke and display decreased expression of Wnt-targeted genes such as *Cldn5* in brain endothelial cells while expression of activated β-catenin restores BBB integrity (Chang et al., 2017).

Endothelial cell heterogeneity and the dynamics of endothelium responses to the changing microenvironment constitute a main constrain to fully study its function, particularly during disease (Aird, 2005). Currently, advances in single-cell transcriptomics are contributing to deeper analysis of EC heterogeneity and to further understanding its dysfunction during disease (Schaum et al., 2018; Zhao et al., 2018; Khan et al., 2019; Kalucka et al., 2020; Jeong et al., 2022; Jones et al., 2022). A single-cell study of the normal and malformed human brain vasculature identified specific endothelial gene expression signatures in arteries, capillaries, venules and veins by using previously defined gene markers of these different vasculatures (Winkler et al., 2022). Pro-inflammatoy, pro-angiogenic and pro-permeability genes were upregulated in the ECs of arteriovenous malformations, which correlated with increased number of CD8^+^ T cells and myeloid immune cells in the perivascular space (Winkler et al., 2022).

Endothelial cells express several innate immune receptors that recognize blood-born factors contributing to immune responses through several mechanisms (Pais and Penha-Gonçalves, 2019). Classical functions include, 1) regulation of innate and adaptive immune cells recruitment and extravasation from the circulation into the tissue parenchyma, through differential expression of adhesion molecules and chemokines; and 2) antigen presentation that is mostly restricted to subsets of ECs with capacity to capture antigen and processing it for presentation, thus performing as semi-professional antigen presenting cells (APC) (Pober and Sessa, 2007; Amersfoort et al., 2022). More recently, tissue-specific subsets of ECs have been observed to modulate the immune response in particular organs (Amersfoort et al., 2022). Murine and human single-cell transcriptional analysis enabled the transcriptomic profiling of EC subsets with specialized biological features. Interestingly, a population of capillary ECs expressing interferon-response genes (*Ifit1*, *Ifit3*, *Ifit3b*, *Igtp*, *Isg15*, *Stat1*, *Tgtp2*, *Usp8*), and thus resembling an “interferon-activated EC population,” was identified in healthy mouse organs, namely, brain, heart, muscle and spleen (Kalucka et al., 2020). The authors of this study hypothesize that this particular EC population might play a role in immunological surveillance. These findings also point to a high degree of heterogeneity in the activation of type I IFN in brain ECs, which requires further investigation.

## 3 Type I IFN response and IFNAR signaling

Interferon (IFN) was originally coined to describe a cell-released “interfering agent” that inhibited influenza virus growth in hen’s eggs (Isaacs and Lindenmann, 1957). Type I IFNs, however, have been linked to immunomodulatory, antiproliferative and neurologic functions [reviewed in (Crow et al., 2019)]. IFN-α, IFN-β, IFN-ε, IFN-κ and IFN-ω are all members of the type I IFN gene family, clustered in the human chromosome 9 and mouse chromosome 4. The biological activities of IFNα (13 subtypes in humans and 14 in mice) and IFNβ are mostly studied because infection induces secretion of these particular interferons in high amounts [reviewed in (Fox et al., 2020)].

The detection of secreted type I IFNs is hindered by the low sensitivity of currently available ELISA-based assays. On the other hand, type I IFN signaling is usually indirectly measured by the expression of genes induced upon Type I IFN stimulation, the so-called IFN- stimulated genes (ISGs). The generation of reporter mouse strains such as the IFNβ-luciferase reporter mice, which express luciferase under the control of the IFN-β promoter, allowed for the tracking of IFNβ producer cells *in vivo* and offer a direct measure of IFNβ expression levels in different tissues (Lienenklaus et al., 2009).

Interestingly, IFNα was the first biotherapeutic approved by regulatory agencies in the 1980s, and it was initially used to treat a type of leukemia and liver viral infections. IFNβ was approved in 1993 as a treatment for the relapsing-remitting form of multiple sclerosis (Borden et al., 2007).

### 3.1 IFNAR signaling

One of the most intriguing aspects of the type I IFN response is that all type I IFNs bind to and signal through the same receptor: the type I IFN receptor (IFNAR) that is ubiquitously expressed. IFNAR is composed of the high-affinity binding subunit IFNAR2 and the signaling transduction subunit IFNAR1. The associated Janus activated kinase 1 (JAK1) and thyrosine kinase 2 (TYK2) undergo autophosphorylation upon type I IFN binding and activate the JAK-STAT (“signal transducer and activator of transcription”) signaling pathway [reviewed in (Ivashkiv and Donlin, 2014)]. The IFN-stimulated gene factor 3 (ISGF3) complex is assembled by phosphorylated STAT1 and STAT2, as well as by the interferon regulatory factor 9 (IRF9). ISGF3 acts as a transcriptional factor that binds to IFN-stimulated response elements (ISREs) and induces ISGs expression (e.g., *Mx1*, *Ifit1*, *Isg15* and *Usp18*) [reviewed in (Schneider et al., 2014)]. Several mechanisms contribute to IFNAR-mediated responses upon stimulation by type I IFN molecules: 1) the different binding affinities to the receptor of different type I IFN molecules (Fox et al., 2020); 2) the assembling of different STAT complexes (homodimers and heterodimers of STAT3, 4, 5 and 6); 3) the activation of additional signaling pathways (e.g., PI3-AKT, p38 and ERK); 4) the priming of type I IFN response by different cytokines (e.g., IFNγ, IL1β and IFNβ) and 5) the negative regulation of type I IFN signaling [reviewed (van Boxel-Dezaire et al., 2006)].

### 3.2 Induction of type I IFN

The induction of type I IFN is primarily mediated by innate pathogen recognition receptors (PRRs) known to detect nuclei acids [reviewed in (Gazzinelli et al., 2014)]. Endosomal membrane Toll-like receptors (TLRs) detect RNA (TLR3, 7 and 8) and DNA (TLR9) from bacteria and viruses exposed in the lumen of endosomes and lysosomes. Retinoic acid inducible gene (RIG) I-like receptors, including the melanoma differentiation associated gene 5 (MDA5) and RIG I receptors, are activated by synthetic or viral-derived double-stranded RNA (dsRNA) present in the cytosol. After binding the RNA, the caspase activation and recruitment domains (CARDs) of these cytosolic receptors interact with a CARD domain of the outer mitochondrial antiviral signaling (MAVS) protein. Upon MAVS oligomerization, TNF receptor-associated factor (TRAF) proteins are recruited to form the “MAVS signalosome”. This complex then initiates a signaling cascade that results in phosphorylation of the type I IFN transcription factors IRF3 and IRF7 by TANK-Binding Kinase 1 (Refolo et al., 2020).

The enzyme cyclic-GMP-AMP (cGAMP) synthase (cGAS) binds to cytosolic dsDNA. As a result, cGAS undergoes catalytic pocket changes and synthesizes 2′3′-cyclic guanosine-adenosine mono phosphate (2′3′-cGAMP) [reviewed in (Wan et al., 2020)]. 2′3′-cGAMP functions as a second messenger and activates the stimulator of IFN genes (STING) receptor, also known as stimulator of interferon response cGAMP interactor 1 (STING1) or TMEM173. A critical step in type I IFN induction is the trafficking of STING from the endoplasmic reticulum (ER) to the Golgi compartments, which is triggered by interaction of 2′3′-cGAMP with STING [reviewed in (Ergun and Li, 2020)]. The cGAS-STING pathway is critical for the response to cellular damage that misplaces mitochondrial and genomic dsDNA, and for the response to infection by detecting microbial dsDNA. Activation of the cGAS-STING pathway by self-DNA in non-hematopoietic cells such as the endothelium seems to contribute to the pathophysiology of several autoinflammatory and neurodegenerative disorders [reviewed in (Skopelja-Gardner et al., 2022)].

### 3.3 STING

In addition to being induced upon dsDNA sensing via the cGAS-STING pathway, type I IFN responses can be triggered by cGAS-independent STING activation. STING is a transmembrane ER protein with activation binding site in the homodimer’s cytosolic domain. STING dimers undergo a conformational change in response to cGAMP binding, which “closes” the dimer and facilitates the recruitment of additional STING molecules [reviewed in (Ergun and Li, 2020)]. In addition to 2′3′-cGAMP, cyclic di-AMP secreted by bacteria directly binds STING, albeit with a lower binding affinity [reviewed in (Cheng et al., 2022)]. Interestingly, STING bystander activation may occur by cGAMP transferred from nearby DNA-responder cells (Ablasser et al., 2013). One of the identified cGAMP intercellular transporters is the SLC19A1, known as a ubiquitously expressed high-affinity importer of reduced folates (Ritchie et al., 2019). After cGAMP binding, the cGAMP-STING complex translocates to the Golgi compartment via the ER-Golgi intermediate compartment (ERGIC) and the vesicle coat protein COP-II (Gui et al., 2019). The STING C-terminal tail (CTT), comprising its last 40 aminoacids, is required for TBK1/IKKε recruitment at the Golgi (Liu et al., 2022). Interestingly, the CTT domain seems to be present only in vertebrates while the cyclic dinucleotide binding domain is evolutionary conserved and present in sea anemones (Kranzusch et al., 2015).

Palmitoylation, a posttranslational modification critical for STING signaling, also occurs in the Golgi compartment (Mukai et al., 2016). Binding of TBK1 to STING oligomers causes autophosphorylation of TBK1, which further phosphorylates the tails of neighboring STING molecules, forming a docking site for TBK1 to phosphorylate IRF3 (Zhang et al., 2019). Subsequent translocation of IRF3 phosphorylated dimers to the nucleus activates the transcription of type I IFN and of other pro-inflammatory cytokine genes (Liu et al., 2015). In addition, STING activation has been demonstrated to activate NF-kB and induce pro-inflammatory genes (TNF, IL1β, and IL-12p40) in macrophages independent of TBK1 (Balka et al., 2020). STING can be transported back to the ER through COP-I vesicles or be recruited to the lysosomes, where it is degraded (Taguchi et al., 2021).

Remarkably, disruption of STING post-Golgi trafficking induces tonic type I IFN signaling even in the absence of pathogenic exposure to DNA. After deletion of the trans-Golgi coiled coil protein GCC2, which regulates Golgi-exit and STING degradation by the lysosome, STING remained in the ER and Golgi. This was sufficient to sustain phosphorylated STING, TBK1 and IRF3 levels in fibroblasts (Tu et al., 2022), which nevertheless require basal cGAS activity to induce ISGs. In fibroblasts and bone marrow-derived macrophages, however, blocking STING lysosomal degradation could promote cGAS-independent ISG expression (Chu et al., 2021). The recently proposed “basal flux” model of STING activation suggested that trafficking interruption of STING can activate type I IFN signaling (Jeltema et al., 2023). According to this model, cell trafficking defects causing protein aggregation in neurodegenerative diseases may also contribute to constitutive activation of STING and disease-associated pathology.

STING drives authophagosome formation and several forms of cell death in addition to the canonical STING pathway of type I IFN and pro-inflammatory cytokines induction [reviewed in (Zhang et al., 2021)]. cGAMP-stimulated autophagosome formation occurs in STING-enriched ERGIC areas, accompanied by LC3 recruitment and lipidation (Gui et al., 2019). Authophagy induction is independent of the CTT STING domain suggesting that this ancient mechanism, also with antiviral function, precedes type I IFN induction in vertebrates (Gui et al., 2019). In mice with a mutation that impairs STING-dependent type I IFN induction, STING activation lead to T cell death, and affected T cell-mediated tumor immunity (Wu et al., 2020). In contrast, another study showed that apoptosis required IRF3 and type I IFN activation in primary human T cells, whereas inhibition of T cell growth was IRF3-independent (Kuhl et al., 2023; Kuhl et al., 2023).

In conclusion, STING activation operates both by type I IFN-dependent and independent mechanisms, with diverse effects in the immune response. In this review, we address the type I IFN signaling pathway in endothelial cells associated with inflammation and BBB dysfunction. Based on recent research, we discuss the role of STING activation in endothelial cells, in type I IFN production, and in inflammatory responses, a rapidly expanding field of study. Nonetheless, there is significant evidence that non-canonical STING activation is involved in the pathogenesis of several diseases, independently of type I IFN production. (Wu and Yan, 2022).

## 4 Type I IFN signaling in the endothelium

Activation of type I signaling in endothelial cells may be triggered by infection but it is also caused by gene mutations, response to cellular stress and changes in inflammatory and ageing-related circulating factors (Chen et al., 2020). The type I IFN response in the endothelium may have a major impact on organ function by increasing vascular permeability, inflammatory cell recruitment, and angiogenesis.

### 4.1 Interferonopathies

Interferonopathies are inherited monogenic autoinflammatory disorders characterized by overactivation of type IFN signaling. Since 1984, when Jean Aicardi and Françoise Goutières described the first interferonopathy, 38 identified gene mutations have been associated to interferonopathies [reviewed in (Crow et al., 2022)].

The Aicardi–Goutières syndrome (AGS) was defined as a familial encephalopathy of non-viral origin with increased levels of IFNα in the serum and in cerebral spinal fluid. The majority of interferonopathies are caused by gene mutations responsible for cytosolic accumulation of nucleic acids, which promotes innate immune responses against self-DNA via activation of type I IFN signaling. Interferonopathies are phenotypically heterogeneous with a wide range of clinical features, but the majority share neurological manifestations. Furthermore, cerebral and skin vasculopathy (d’Angelo et al., 2021; Xin et al., 2011) are common, highlighting that abnormal type I IFN responses target endothelial cells. In particular, STING-associated vasculopathy with onset in infancy (SAVI) is caused by *de novo* mutations in TMEM173, the gene encoding STING, and is associated with a type I IFN response signature and increased blood levels of CXCL10 chemokine, a well-known ISG (Liu et al., 2014). SAVI syndrome is distinguished by the early-onset of systemic inflammation in children, manifested by severe cutaneous vasculopathy and pulmonary inflammation. Inflammatory neutrophil and T-lymphocyte infiltrates are found around damaged vessels, some of which are occluded by fibrin deposits. Endothelial inflammation, intravascular coagulation and enhanced expression of adhesion molecules by vascular endothelial cells were reported in SAVI (Liu et al., 2014).

### 4.2 TNF-dependent type I IFN signaling

The primary target of circulating TNF during inflammation is the endothelium. TNF promotes expression of cell adhesion molecules (CAMs) on the vascular endothelium and production of chemokines. These responses contribute to the recruitment, activation and transmigration of leukocytes across the endothelium (Amersfoort et al., 2022). TNF association with type I IFN signaling was first uncovered by its antiviral activity in hepatocytes (Mestan et al., 1988). Only after 20 years was the mechanism found in TNF-activated human macrophages (Yarilina et al., 2008). TNF activates the IRF1 transcription factor, which together with NF-kB and AP1 results in IFNβ production. In turn, IFNβ acts in an autocrine manner via IFNAR, activating STAT1 and type I IFN response genes such as the chemokine CXCL10 (Yarilina et al., 2008). IFNAR1 signaling is also required *in vivo* during TNF-induced lethal shock (Huys et al., 2009).

In endothelial cells, TNF also stimulates the same IRF1-IFNβ-IFNAR-STAT1 autocrine loop through TNFR2 and TNFR1. In mice, intravenous injection of soluble TNF induces STAT phosphorylation and the expression of CXCL10 protein in glomerular endothelial cells, which is required for kidney monocyte recruitment (Venkatesh et al., 2013). The role of type I IFN in leukocyte recruitment was further demonstrated in a mouse model of TNF-induced peritonitis. Upon TNF treatment, IFNAR^−/−^ mice have lower recruitment of leukocytes (CD45^+^ cells) including CD4^+^ T lymphocytes into the peritoneal cavity as compared to wild-type mice (Anastasiou et al., 2021). Interestingly, T lymphocyte recruitment mediated by IFNAR signaling depended on endothelial-specific STING expression. TNF stimulated endothelial cells induced T cell transendothelial migration (TEM) in a STING-dependent manner *in vitro*. STING activation resulted in type I IFN production, which promoted T cell TEM by inducing endothelial CXCL10 expression via the IFNAR-JAK/STAT axis. Adhesion molecules such as VCAM-1, ICAM-1, and PECAM-1, on the other hand, did not differ in expression between wild-type and STING-deficient endothelial cells when stimulated by TNF (Anastasiou et al., 2021; Pais et al., 2022), which indicates that T cell adhesion to the endothelium was unaffected.

Another possibility, as described in human monocytes, is that increased mitochondrial DNA in the cytosol due to impaired mitochondrial function causes a type I IFN response via the cGAS-STING pathway in TNF-activated endothelial cells (Willemsen et al., 2021). This induction of type I IFN via TNF response may play an important role in endothelial response to infection and chronic inflammation.

### 4.3 Type I IFN response in brain endothelial cells

**FIGURE 1:**
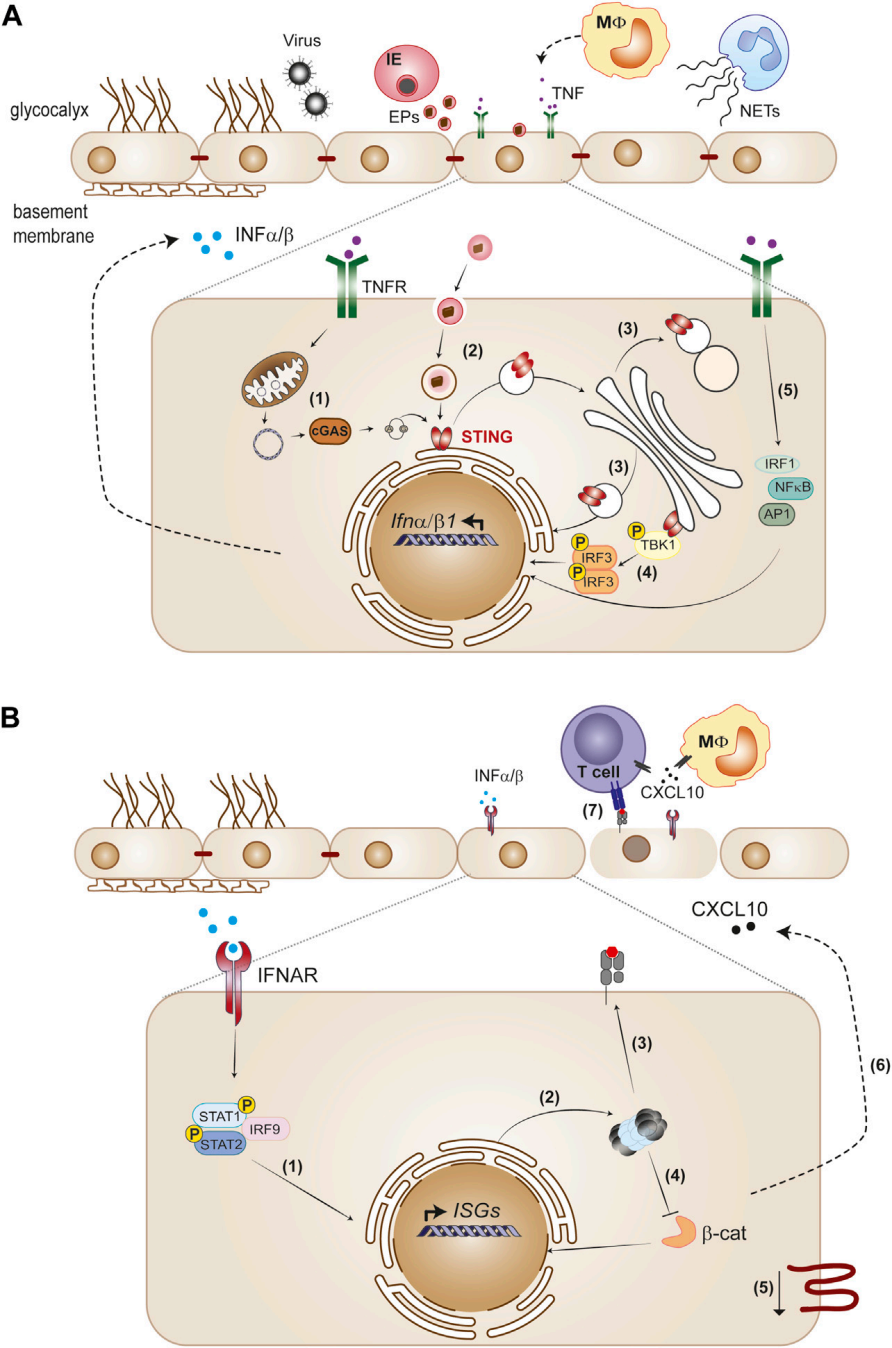
The endothelium as initiator and target of type I IFN responses. **(A)** Viruses and *Plasmodium*-infected erythrocytes (IE), neutrophil extracellular traps (NETs) and cytokines such as TNF released by monocytes trigger type I IFN in the endothelium. STING activation is a main pathway of IFNα/β gene transcription induction in the endothelium. Cellular stress produced by infection or TNF causes leaky mitochondrial DNA that binds cGAS and generates 2′3′-cGAMP, which activates STING (1). Extracellular particles (EPs) derived from IE (2), as well as defects in STING trafficking across endoplasmatic reticulum, Golgi and lysosomal compartments (3) also activate the STING pathway. STING recruits TBK1 in the Golgi, which phosphorylates IRF3 and induces IFNβ transcription (4). TNFR signaling induces IFNβ through IRF1, in cooperation with other TNF-activated transcription factors, NF-κB and AP1 (5). **(B)** Paracrine IFNAR1 signaling induction by endothelial secreted IFNα/β activates the phosphorylated STAT1/STAT2 dimer with IRF9 complex that migrates to the nucleus and directs ISGs expression (1). Type I IFN signaling induces immunoproteasome activation (2) and promotes MHC class I antigen presentation (3). Immunoproteasome activation inhibits the Wnt/β-catenin signaling pathway (4), which is associated with downregulation of tight junction components (5). On the other hand, type I IFN signaling induces CXCL10 secretion (6), leading to leukocyte recruitment to the endothelium, namely, CD4^+^ and CD8^+^ T cells. Activation of CD8^+^T cytotoxic cells through interaction with MHC class I peptide complexes on the endothelium layer combined with decrease of tight junction proteins contribute to loss of endothelial barrier function (7).

Brain endothelial cells activate innate immune mechanisms in response to systemic alterations, which may have both deleterious and beneficial impact in brain functioning (Pais and Penha-Gonçalves, 2019). Among these innate responses, type I IFN has been linked to cognitive impairment and neuroinflammation in virus-induced sickness (Blank et al., 2016), Aicardi-Goutières syndrome (d’Angelo et al., 2021), stroke (Kang et al., 2020), brain injury (Abdullah et al., 2018), multiple sclerosis (MS) (Raftopoulou et al., 2022) and neurodegenerative diseases such as Alzheimer’s disease (AD) (Roy et al., 2022). In addition, type I IFN plays a role in CNS homeostasis (Goldmann et al., 2016). The activation of type I IFN signaling in the brain endothelium could represent an initial response to pro-inflammatory factors in circulation, which can subsequently be amplified by other brain cells (Owens et al., 2014). On the other hand, production of type IFN by CNS resident cells (Raftopoulou et al., 2022) can also activate IFNAR receptor on brain endothelium. Understanding the type I IFN response in brain endothelial cells may identify important cues in the cross-talk between the periphery and CNS (Figure 1).

Type I IFN signaling in brain endothelial cells has primarily been studied in multiple sclerosis (MS) and viral infection throughout the years, with both beneficial and deleterious effects on the BBB. Administration of IFNβ is one of the most successful treatments of MS. The main therapeutic benefit resides in IFNα/β-mediated suppression of pathogenic T cells, but *in vitro* studies show an additional direct impact of interferons on the BBB. IFNβ inhibits both the expression of adhesion molecules and the transendothelial migration of monocytes (Floris et al., 2002). Furthermore, IFNβ prevents the loss of BBB function caused by astrocyte depletion or by incubation with histamine (Kraus et al., 2004). Similarly, West Nile virus (WNV) and mouse hepatitis virus (MHV) neuroinvasion are prevented by viral-induced IFNβ, which reinforces endothelial tight junctions and improves its barrier function (Daniels et al., 2014).

In part, type I IFN effects on the BBB were linked to the activity of Rho GTPases, a family of signaling molecules that affects tight junction stability and paracellular permeability by controlling cytoskeletal dynamics (Beckers et al., 2010). In an *in vitro* BBB model, TNF and IL-1β activate RhoA, which impairs the barrier function and increases West Nile virus transendothelial migration. IFNβ, on the other hand, decreases RhoA activity while increasing Rac-1 thereby stabilizing the BBB (Daniels et al., 2014). Animal studies have also shown that type I IFN protects the BBB. Mice pre-conditioning with poly I:C, a synthetic dsRNA, elicits IFNAR signaling in the brain endothelial cells and protects against ischemia-induced brain injury (Gesuete et al., 2012). MRI studies also show that systemic administration of IFNβ after stroke reduces vasogenic edema, leukocyte infiltration and brain volume lesion in rats (Veldhuis et al., 2003). In contrast, recent studies found that IFNβ has a negative impact on the brain endothelium. Administration of IFNβ into brain lesions after mild traumatic brain injury prevents meningeal vascular repair (Mastorakos et al., 2021). Interestingly, DNA in neutrophil extracellular traps (NETs) activate the cGAS-STING pathway inducing IFNβ and cerebrovascular complications after stroke (Kang et al., 2020; Wang et al., 2021). In this setting, IFNβ in the brain caused tight junction disruption and BBB permeability, although it is unclear whether IFNβ induction or IFNAR signaling were directly triggered in brain endothelial cells. DNAse treatment and STING silencing both reversed this detrimental effect and promoted the vascular remodeling following stroke (Kang et al., 2020).

Further supporting a type I IFN detrimental effect on the brain vasculature, MDA5-dependent type I IFN induction by viral infection delayed BBB repair in models of post traumatic brain injury (TBI) and cerebrovascular injury. (Mastorakos et al., 2021). Activation of IFNAR signaling in the myeloid cell compartment is required for this effect, while it is unclear if downstream IFNAR signaling on brain endothelial cells leads to vascular impairment. In a transgenic mouse model of AD, a type I IFN signature in brain endothelial cells was linked to increased BBB permeability and amyloid-beta deposition (Jana et al., 2022). This endothelial response could be secondary to the disease itself, as activation of IFNAR signaling specifically in microglia and neurons drives neuropathology in this AD mouse model (Roy et al., 2022). Recently, analysis of post-mortem brain tissue from MS and AD patients showed colocalization of calnexin, a marker of activated endothelial cells, with STING in brain capillaries (Ferecskó et al., 2023). The authors show that activation of human brain endothelial cells with palmitic acid, an inflammatory agent in neurodegenerative diseases, leads to the release of mitochondrial DNA resulting in STING activation, IRF3 phosphorylation and IFNβ production.

Although several neurologic conditions activate type I IFN signaling in the brain endothelium, the connection to the pathology is not always clear.

Cerebral malaria (CM), a severe form of malaria, is a neurologic syndrome caused by *Plasmodium falciparum* infection with a mortality of up to 25% even among hospitalized and treated children (Idro et al., 2010; Savonius et al., 2023). CM develops during the parasite blood stage of infection. It involves different pathogenesis mechanisms that dependent on host factors and parasite virulence, and culminate in the loss of BBB function (Pais and Penha-Gonçalves, 2019). By using an experimental mouse model of CM, we have shown that STING activation in endothelial cells contributes to brain accumulation of leukocytes including activated CD8^+^T cells that drive BBB damage during CM, via the IFNβ-CXCL10 axis (Pais et al., 2022). Concomitantly, IFNAR autocrine/paracrine signaling in brain endothelial cells promoted immunoproteosome activation and enhanced antigen presentation by MHC Class I upon exposure to malaria parasite-infected erythrocytes (Shafi et al., 2023). This may help parasite-specific CD8^+^ cytotoxic T lymphocytes kill activated brain endothelium and contribute to BBB disruption during cerebral malaria (Howland et al., 2013). In addition, IFNAR signaling and immunoproteosome activation in endothelial cells autonomously contribute to increase the permeability of the brain endothelial barrier via inhibition of the Wnt/β-catenin signaling pathway (Shafi et al., 2023) (Figure 1).

Therefore, many factors may influence the different cellular downstream effects and pathological outcomes of type I IFN signaling in the brain endothelium (Table 1). These include the response to locally produced type I IFN or to systemically available/administered type I IFN; the cell source and cell target of type I IFN; the nature of stimuli (infectious context or sterile origin); the particular PRR that are activated; and the presence of other immune cells and inflammatory cytokines.

**TABLE 1:** Effects of type I IFN signaling in the brain endothelium.

Disease/model	Stimulus	Pathway	Effects	Ref
AGS	*SAMHD1* mutations	Unknown	Structural changes	Xin et al. (2011)
Stroke	NETs (DNA)	cGAS-STING	BBB dysfuntion	Kang et al. (2020), Wang et al. (2021)
Brain injury-associated infections	LCMV infection, LPS	IFNAR-MDA5	Vascular repair inhibition	Mastorakos et al. (2021)
Alzheimer’s disease	Amyloid-beta deposition	ISG activation	BBB dysfunction	Jana et al. (2022)
Cerebral malaria	EPs of *Plasmodium*-IE	STING	Leukocyte recruitment	Pais et al. (2022)
			BBB dysfunction	
CNS viral infection	PRR activation in BBB	IFNAR-IRF7	BBB stability	Daniels et al. (2014)
*In vitro* BBB	IFNβ	Rho GTPases	BBB stability	Daniels et al. (2014)
	*Plasmodium*-IE	IFNAR	BBB dysfunction	Shafi et al. (2023)
Brain endothelial cells	*Plasmodium*-IE	IFNAR	Immuno-proteosome activation	Shafi et al. (2023)
			Antigen-presentation	

Abbreviations: AGS, Aicardi–Goutières syndrome; SAMHD1, SAM, And HD, Domain Containing Deoxynucleoside Triphosphate Triphosphohydrolase 1; NETs, neutrophil extracellular traps; LCMV, lymphocytic choriomeningitis virus; BBB, blood-brain barrier; EPs, extracellular particles; IE, infected erythrocytes.

### 4.4 STING-mediated vasculopathy in cerebral malaria and COVID-19

The coronavirus-19 (COVID-19) pandemic caused by the severe acute respiratory syndrome coronavirus-2 (SARS-CoV-2) ignited intensive research on the underlying immunopathology mechanisms [reviewed in (Rybkina et al., 2022)]. COVID-19 and severe malaria share a hyperinflammatory response (“cytokine storm”) and several clinical manifestations, which complicates correct diagnosis and treatment of COVID-19 patients in endemic malaria regions (Chaturvedi et al., 2022). Understanding the similarities in type I IFN responses between CM and COVID-19 may elucidate general mechanisms of type I IFN responses in infectious diseases. In both CM and COVID-19, the timing of type I IFN induction appears to be particularly important. The involvement of STING in the vasculopathology, the possibility of a synergistic effect between the type I IFN response and TNF, and the role of type I IFN in the long-term cognitive deficits are also shared features of type I IFN responses to these infectious diseases.

**FIGURE 2:**
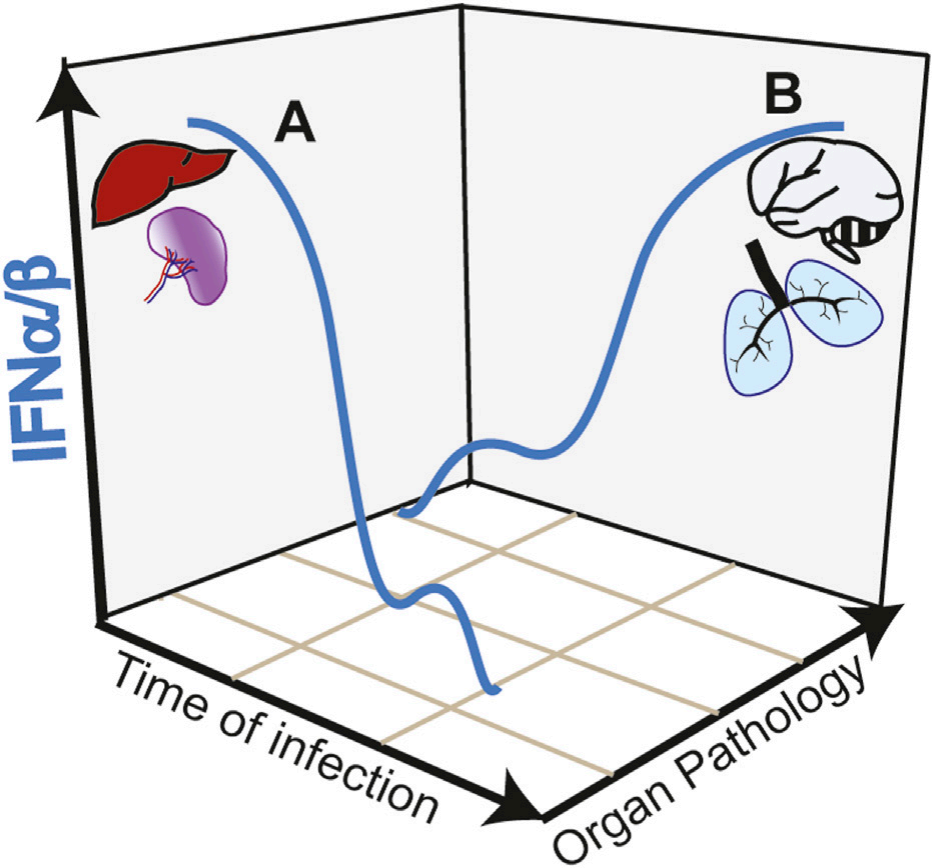
Trajectories of type I IFN responses and organ pathology in infectious diseases. **(A)** Early and strong type I IFN responses in the spleen and liver induce anti-parasite immunity during *Plasmodium* infection avoiding organ targeting and protecting from cerebral pathology (CM). Similarly, in COVID-19, early induction of type I IFN controls viral load preventing lung pathology. **(B)** Early low intensity type I IFN responses, allow pathogen expansion. A second wave of type I IFN, induced by STING activation in the endothelium acts in paracrine fashion causing hyperinflammation of target organs such as the brain and the lung in CM and COVID-19, respectively.

While the initial type I IFN response appears protective in SARS-CoV-2 infection and in CM, activation of this pathway in target organs such as the lung and the brain is highly deleterious. The anti-viral activity of type I IFN response against SARS-CoV-2 is underlined by increase severity of COVID-19 manifestations in patients with impaired type I IFN response (Smith et al., 2022). These impairments are in some cases associated with genetic defects, specifically in the TLR7 gene (Fallerini et al., 2021) or with neutralizing autoantibodies against type I IFN (Bastard et al., 2020). In a mouse model of SARS-CoV-2 infection, disrupting IFNAR signaling increased viral load and disease severity (Ogger et al., 2022). Similarly, in CM, upregulation of type I IFN is linked to protective immunity in children with uncomplicated malaria (Krupka et al., 2012; Boldt et al., 2019). Early induction of a strong type I IFN in the liver and spleen promote anti-parasitic immune responses avoiding brain pathology in experimental CM models [reviewed in (Sebina and Haque, 2018)] (Figure 2, trajectory A of IFNα/β production during infection). IFNα treatment of mice immediately after infection (Vigário et al., 2007) or co-infection with a virus that induces an early type I IFN response (Hassan et al., 2020) reduces parasitemia and confers resistance to CM.

In contrast to the early protective effect, research on COVID-19 showed that a late and sustained type I IFN response, most likely caused by high viral loads, was associated with severe disease outcomes (Figure 2, trajectory B of IFNα/β production during infection). This response occurs in the lower respiratory airways and is associated with tissue damage and apoptosis in the lungs of patients with severe COVID-19 (Sposito et al., 2021). Induction of ISGs, which include monocyte recruitment chemokines like *Cxcl10*, *Ccl7* and *Ccl2* drives the recruitment of inflammatory cells into lungs of SARS-CoV-2-infected mice (Israelow et al., 2020). In contrast to the anti-viral activity mediated by TLR3 and TLR7, this second wave of type I IFN response is associated with cGAS-STING activation. Post-mortem analysis of lungs from COVID-19 patients with a rapid lethal course revealed phosphorylation of STING in the lung’s macrophages and endothelial cells (Domizio et al., 2022). In a lung-on-chip model that mimics the alveolar-capillary interface, alveolar epithelial cells are cultured on the top channel of the microfluidic device separated by a membrane from endothelial cells facing the bottom channel*.* SARS-CoV-2 infection of alveolar epithelium induces STING phosphorylation and IFNβ in endothelial cells but not in the epithelial cells. In addition, endothelial cells undergo cell death. Both IFNβ production and cell death could be blocked by the STING inhibitor H-151 in the vascular side or by STING knockdown in the endothelial cells. Mitochondrial stress with release of endogenous mitochondrial DNA was upstream the cGAS-STING activation pathway and IFNβ induction in endothelial cells, which could be inhibited by blocking mtDNA release (Domizio et al., 2022). However, it is not clear whether IFNβ was required to induce endothelial cell death in this experimental setting. Endothelial cell death was induced, independently of virus replication in endothelial cells and of RNA-sensing RIG-I like receptors. In SARS-Cov-2 infected mice, inhibiting STING reduces lung pathology and inhibits cell death and type I IFN signaling without affecting the viral load (Domizio et al., 2022). Overall, data suggest that PRRs play different roles in different cell types during the course of COVID-19 pathology (Chiale et al., 2022).

Polymorphisms in regulatory regions of type I IFN receptor gene (IFNAR1 subunit) was associated with increased expression of type I IFN, enhanced type I IFN signaling and susceptibility to CM in children (Feintuch et al., 2018). Mice lacking IFNAR1 (Ball et al., 2013), the transcription factors interferon regulatory factor 3 (IRF3) and 7 (IRF7), or the upstream TAK-binding kinase 1 (TBK1), were protected from neurologic symptoms, BBB leakage and death by CM providing compelling evidence of type I IFN-mediated brain pathology in CM (Sharma et al., 2011). Recently, through the use of cell-specific IFNβ-reporter mice, we have identified myeloid (monocytes and microglia) cells and endothelial cells as the IFNβ producing cells in the brain of *Plasmodium*-infected mice (Pais et al., 2022). We found that the recruitment of leukocytes into the brain is specifically determined by STING-dependent IFNβ induction in the brain endothelium via induction of *CXCl10* (Figure 2, trajectory B of IFNα/β production during infection). STING in endothelial cells was activated following the uptake of heme-containing particles released by *Plasmodium*-infected erythrocytes. We showed that heme may activate STING through direct interaction but did not rule out the possibility of upstream cGAS activation by mitochondrial DNA. In contrast to the critical role of STING, we showed that activation of other PRRs via MyD88-dependent pathways or through MAVS are not required in the development of CM pathology (Pais et al., 2022).

The involvement of STING in pulmonary inflammation and vasculopathy in COVID-19 prompted the comparison with SAVI disease, suggesting that gene polymorphisms in the STING pathway could also be associated with COVID-19 (Berthelot and Lioté, 2020). It would be interesting to analyze if STING gene polymorphism´s are associated to CM susceptibility as well. The induction of a type I IFN response in conjunction with TNF is another intriguing aspect that may contribute to the immunopathology of both COVID-19 and CM. Interestingly, postmortem lung tissues from patients with lethal COVID-19 as well as PBMCs from severe COVID-19 cases, show a type I IFN response transcriptional signature, along with TNF/IL-1β induction (Lee et al., 2020). It is possible that type I IFN and TNF responses may synergize also in CM. In fact, TNF was shown to contribute to vasculopathy in CM (Rudin et al., 1997; Zuniga et al., 2022) and to induce a type I IFN response in endothelial cells (Venkatesh et al., 2013).

As suggested for type I IFN-dependent COVID-19 cognitive symptoms (Suzzi et al., 2023), we hypothesize that the type I IFN responses, namely, those involving the brain endothelium, may underlie neurological sequelae in children who survive CM. Activation of type I IFN signaling pathway in the CNS has been linked to cognitive impairment in diseases such as interferonopathies, viral infections, Alzheimer’s disease (Roy et al., 2020) and age-related inflammation (Baruch et al., 2014; Deczkowska et al., 2017), as well as IFNα/β therapies (Valentine and Meyers, 2005). Despite persistent cognitive impairment in COVID-19 patients, there is no strong evidence that SARS-CoV-2 infects the brain. The choroid plexus epithelium, on the other hand, appears to respond to infection by increasing genes associated with type I IFN signaling and cell communication with oligodendrocytes, microglia and neurons through CCL and CXCL family of chemokines (Yang et al., 2021). High levels of chemokines, including CCL2, CCL11, and CXCL10, were found in the cerebral spinal fluid (CSF) of the mouse model of mild COVID-19 and persisted during infection. This correlates with increased microglial reactivity and decreased hippocampal neurogenesis (Fernández-Castañeda et al., 2022). These observations support the possibility that cognitive risks in COVID-19 infection may be promoted by persistent low-grade inflammation driven by IFN-chemokines signaling loops involving the choroid plexus epithelium, microglia and astrocytes, as proposed by (Suzzi et al., 2023). CXCL10 may also act on neurons. CXCL10 induction by IFNAR signaling in brain endothelial cells has been shown to promote virus-induced sickness behavior by binding to CXCR3 on neurons and changing synaptic plasticity (Blank et al., 2016). We hypothesize that during CM a similar mechanism may operate and contribute to the neurologic symptoms and cognitive impairment in patients surviving the infection.

## 5 Conclusion and perspectives of STING targeting in the endothelium

Protective, pathological and therapeutic effects of type I IFN are context-dependent and are highly influenced by the nature of innate stimuli, the innate receptors that are engaged, the responding genes (ISGs) and the cell types involved in type I IFN secretion and signaling, as well as the inflammatory milieu including the action of other inflammatory factors, namely, TNF. However, type I IFN responses associated with cGAS-STING activation appear to be critical in promoting organ inflammation and immune-mediated damage. Given the potential clinical applications in inflammatory diseases, efforts have been made to develop small-molecule inhibitors of the cGAS-STING pathway (Decout et al., 2021). We hypothesize that inhibiting this pathway at the endothelium level may be an effective approach to minimize auto-inflammatory events and infection-driven immunopathology in organs such as the brain and lung. On the other hand, drugs activating STING in endothelial cells may help to target cancer tissue increasing the efficiency of cytotoxic adaptive immune responses. It has been proposed that intratumoral injection of cGAMP enhance STING activation on endothelial cells and strongly promote the generation of CD8^+^ T cell responses that efficiently control the growth of the injected tumor and of contralateral tumors (Demaria et al., 2015).

Although these approaches may face pharmacological challenges related to drug delivery, tissue-specificity and off-target effects, the strategy of modulating type I IFN responses by targeting one specific innate sensor may offer a successful path in obtaining defined and unmet therapeutic outcomes.
